# Amelioration of Endotoxemia by a Synthetic Analog of Omega-3 Epoxyeicosanoids

**DOI:** 10.3389/fimmu.2022.825171

**Published:** 2022-02-24

**Authors:** Akira Shikuma, Daisuke Kami, Ryotaro Maeda, Yosuke Suzuki, Arata Sano, Toshihiko Taya, Takehiro Ogata, Anne Konkel, Satoaki Matoba, Wolf-Hagen Schunck, Satoshi Gojo

**Affiliations:** ^1^ Department of Cardiovascular Medicine, Graduate School of Medicine, Kyoto Prefectural University of Medicine, Kyoto, Japan; ^2^ Department of Regenerative Medicine, Graduate School of Medicine, Kyoto Prefectural University of Medicine, Kyoto, Japan; ^3^ Department of Pathology and Cell Regulation, Graduate School of Medicine, Kyoto Prefectural University of Medicine, Kyoto, Japan; ^4^ OMEICOS Therapeutics , Berlin, Germany; ^5^ Max Delbruck Center for Molecular Medicine, Berlin, Germany

**Keywords:** Omega-3, Unsaturated fatty acids, Mitochondria, Macrophage, Inflammation

## Abstract

Sepsis, a systemic inflammatory response to pathogenic factors, is a difficult to treat life-threatening condition associated with cytokine and eicosanoid storms and multi-organ damage. Omega-3 polyunsaturated fatty acids, such as eicosapentaenoic (EPA) and docosahexaenoic acid, are the precursors of potent anti-inflammatory lipid mediators, including 17,18-epoxyeicosatetraenoic acid (17,18-EEQ), the main metabolite of EPA generated by cytochrome P450 epoxygenases. Searching for novel therapeutic or preventative agents in sepsis, we tested a metabolically robust synthetic analog of 17,18-EEQ (EEQ-A) for its ability to reduce mortality, organ damage, and pro-inflammatory cytokine transcript level in a mouse model of lipopolysaccharide (LPS)-induced endotoxemia, which is closely related to sepsis. Overall survival significantly improved following preventative EEQ-A administration along with decreased transcript level of pro-inflammatory cytokines. On the other hand, the therapeutic protocol was effective in improving survival at 48 hours but insignificant at 72 hours. Histopathological analyses showed significant reductions in hemorrhagic and necrotic damage and infiltration in the liver. *In vitro* studies with THP-1 and U937 cells showed EEQ-A mediated repression of LPS-induced M1 polarization and enhancement of IL-4-induced M2 polarization of macrophages. Moreover, EEQ-A attenuated the LPS-induced decline of mitochondrial function in THP-1 cells, as indicated by increased basal respiration and ATP production as well as reduction of the metabolic shift to glycolysis. Taken together, these data demonstrate that EEQ-A has potent anti-inflammatory and immunomodulatory properties that may support therapeutic strategies for ameliorating the endotoxemia.

## Introduction

Omega-3 polyunsaturated fatty acid (n-3 PUFA) supplementation is associated with improved outcomes in patients with sepsis including reduction of mortality, duration of mechanical ventilation, and intensive care unit length of stay, as shown in a recent meta-analysis of randomized clinical trials (RCTs) (1). N-3 PUFA-supplementation is currently also discussed as potential adjuvant therapy in COVID-19 patients to promote resolution of inflammation (2) and to mitigate the development of cardiovascular complications (3). A pilot study suggested that the risk of death from COVID-19 inversely correlates with the red blood cell level of the long-chain n-3 PUFAs eicosapentaenoic acid (EPA) and docosahexaenoic acid (DHA) (4). Currently, at least 14 RCTs with different n-3 PUFA formulations are ongoing to investigate their preventive and therapeutic potential in COVID-19 (5).

Providing a rationale for their use in bacterial sepsis and corona virus-induced hyperinflammation, n-3 PUFAs are the precursors of lipid mediators with potent anti-inflammatory and cell-protective properties. These bioactive lipid mediators include EPA- and DHA-derived epoxyeicosanoids (3, 6) and a family of specialized pro-resolving mediators (7–9).

Epoxyeicosanoids are generated from PUFAs *via* the cytochrome P450 (CYP) epoxygenase pathway and are rapidly further metabolized to inactive or even toxic vicinal diols by the soluble epoxide hydrolase (sEH) (10–12). A series of preclinical studies revealed that transgenic overexpression of CYP epoxygenases as well as genetic or pharmacological sEH inhibition attenuate systemic inflammation and multi-organ damage resulting in reduced mortality in mouse models of lipopolysaccharide (LPS)-induced sepsis (13–16). N-3 PUFA-supplementation may also synergize with sEH inhibition to suppress inflammation as shown in a variety of other models (17).

Dietary n-3 PUFA-supplementation results in the formation of 17,18-epoxyeicosatetraenoic acid (17,18-EEQ) and 19,20-epoxydocosapentaenoic acid (19,20-EDP) as the main EPA- and DHA-derived epoxyeicosanoids in rodents and human (18, 19). 17,18-EEQ was reported to alleviate TNFα-induced lung inflammation and hyperresponsivness in human bronchial explants (20) and guinea pig tracheal rings (21). 19,20-EDP protects cardiomyocytes against LPS-induced cytotoxicity (22). Moreover, 19,20-EDP provided protection against ischemia/reperfusion-injury in isolated perfused murine hearts *via* maintaining mitochondrial function and limiting NLRP3 inflammasome activation (23). Omega-3 epoxyeicosanoids also efficiently attenuate inflammatory reactions in animal models of age-related macular degeneration (24, 25), allergic intestinal inflammation (26), and kidney fibrosis (27) after intraperitoneal injection, potentially by altering immune cell function.

Limiting their direct therapeutic utility, Omega-3 epoxyeicosanoids, such as 17,18-EEQ, are prone to autoxidation, membrane incorporation, and rapid enzymatic metabolism by cyclooxygenases, lipoxygenases, and epoxide hydrolases (6). To overcome these limitations, chemically and metabolically robust synthetic analogs of 17,18-EEQ have been developed by reducing the number of double bonds, replacing the epoxy group by epoxy-bioisosters, and introducing a 3-oxa group (6, 28, 29). One of the first analogs was already successfully used to attenuate laser-induced choroidal neovascularization in mice (25). Currently, 17,18-EEQ analogs are under further clinical development (30) by the OMEICOS Therapeutics GmbH (https://omeicos.com/).

In the present study, we accessed the effects of a 17,18-EEQ analog (EEQ-A) on mortality, organ damage, and pro-inflammatory cytokine transcript level in a mouse model of LPS-induced sepsis. Moreover, we performed *in vitro* experiments with THP-1 and U937 cells to investigate potential effects of EEQ-A on macrophage polarization and mitochondrial function.

## Materials and Methods

### Animal Experiments

All experiments were performed according to the animal experiment guidelines issued by the Animal Care and Use Committee at the Kyoto Prefectural University of Medicine and approved by the Animal Experiment Ethics Committee of the Kyoto Prefectural University of Medicine (approval number M2021-547).

### Mouse Intraperitoneal Administration (IP) of EEQ-A and LPS

Young (8-9 weeks) C57BL/6 mice were housed in the animal facilities at Kyoto Prefectural University of Medicine under specific pathogen-free conditions. EEQ-A was provided by OMEICOS Therapeutics. EEQ-A stock solutions were prepared in DMSO and further diluted in phosphate-buffered saline (PBS, FUJIFILM Wako Pure Chemical Corporation, Osaka, Japan). In the prevention protocol, the mice received 50 ng/g body weight IP injections of EEQ-A suspended in 5 μl/g body weight PBS (n=9, male 5, female 4) or the same amount of vehicle (0.1% DMSO in PBS) as the control group (n=9, male 4, female 5) every 24 hours from -48 hours to 0 hours, and 40-50 mg/kg body weight LPS from *Escherichia coli* O55 (Merck KGaA, Darmstadt, Germany, Lot No. 81275, 81276) resuspended in 10 μl/g body weight PBS at 0 hours resuspended in PBS. In the treatment protocol, the mice received 40-50 mg/kg body weight LPS suspended in 10 μl/g body weight PBS at 0 hours and received 50 ng/g body weight EEQ-A IP injections suspended in 5 μl/g body weight PBS (n=19, male 9, female 10) or the same amount of vehicle as the control group (n=19, male 9, female 10) every 24 hours from 0 hours to 48 hours. Mice were examined continuously for survival until 72 hours after the LPS IP injection.

### Histology and Liver Inflammatory Scores

The heart, lung, liver, right kidney, and colon of mice were collected 8 or 24 hours after the last LPS injection and fixed with 4% paraformaldehyde (FUJIFILM Wako Pure Chemical Corporation).

All tissues were embedded in paraffin, cut into sections, and stained with hematoxylin and eosin (HE) at the Histology Consultation Services (Applied Medical Research Laboratory, Osaka, Japan). Images were visualized and recorded using a BIOREVO BZ-9000 fluorescence microscope (Keyence Corporation, Osaka, Japan). Liver inflammatory scores were assessed based on the severity of necrosis, bleeding, and infiltration in the liver using the method described in a previous report (31). Necrosis: normal = 0, mild (focal piecemeal necrosis) = 1, moderate (continuous necrosis in <50% of focal areas) = 2, and severe (continuous necrosis in >50% in focal areas) = 3. Bleeding: normal = 0, mild (<30% of focal areas) = 1, moderate (30–50% of focal areas) = 2, and severe (>50 of focal areas) = 3. Infiltration: normal=0, mild (2- to 3-fold inflammatory cells) = 1, moderate (3- to 10-fold inflammatory cells) = 2, and severe (>10-fold inflammatory cells) = 3.

### Cell Culture and Inflammation Model

THP-1 cells were cultured in Roswell Park Memorial Institute 1640 medium (RPMI 1640 medium, Thermo Fisher Scientific Incorporated, Waltham, Massachusetts, USA) supplemented with 10% fetal bovine serum (FBS, Thermo Fisher Scientific Incorporated) and 1% penicillin/streptomycin (Thermo Fisher Scientific Incorporated) and incubated at 37°C in a humidified 5% CO_2_ incubator. For the THP-1inflammation model, the cells were seeded in 12-well cell culture plates (Corning Incorporated, Corning, New York, USA) at a density of 5 × 10^5^ cells per well in growth medium containing phorbol 12-myristate 13-acetate (PMA, 10 nM) (at -96 hours). After 48 hours (at -48 hours), the supernatant of the medium was carefully removed to avoid detaching the cells attached to the bottom of the plate and replaced with new medium containing the appropriate concentration of EEQ-A or DMSO (0.1%) as a control. 48 hours later (at 0 hours), LPS (indicated concentration) was added, and the cells were harvested at each time point and used for experiments.

### Gene Expression Analysis

Total RNA was extracted from each sample using TRIzol (Thermo Fisher Scientific Incorporated) and a Directzol RNA MiniPrep Kit (Zymo Research, Irvine, California, USA) with DNase I, according to the manufacturer’s instructions. The qPCR assay was performed by reverse transcribing 100 ng of total RNA using the Prime Script RT Reagent Kit and SYBR Premix Ex Taq (Takara Bio, Shiga, Japan) according to the manufacturer’s instructions. qPCR was performed using a CFX Connect Real-Time PCR Detection System (Bio-Rad). The relative mRNA expression levels were normalized to human *GAPDH* or mouse *Gapdh* expression.

The forward and reverse primer sequences are shown in the **Supplementary Table 1**.

### Mitochondrial Membrane Potential (Δφ)

We harvested the cells 1, 4, 8 or 24 hours after LPS addition. The cells were resuspended at a density of 1 × 10^5^/ml in culture medium containing 1 μl/ml Fixable Viability Dye eFluor 450 (Thermo Fisher Scientific Incorporated), 200 nM MitoTracker Green FM (Thermo Fisher Scientific Incorporated) and 100 nM Image-iT TMRM Reagent (Thermo Fisher Scientific Incorporated) and incubated at 37°C for 30 minutes. After staining, the cells were washed immediately, resuspended in AutoMACS Running Buffer (Miltenyi Biotec), and evaluated using an Attune NxT Flow Cytometer (Thermo Fisher Scientific Incorporated).

In this experiment, we selected live cells gated based on the fluorescence intensity of Fixable Viability Dye eFluor 450, and the numeric value was calculated by dividing the fluorescence intensity of Image-iT TMRM Reagent by the fluorescence intensity of MitoTracker Green FM (TMRM/MitoTracker Green FM) as an index of Δφ.

Measurement of mitochondrial reactive oxygen species (mtROS) levels

We harvested the cells 1, 4, 8 or 24 hours after LPS addition. The cells were resuspended at a density of 1 × 10^5^/ml in culture medium containing 1 μl/ml Fixable Viability Dye eFluor 450 (Thermo Fisher Scientific Incorporated) and 5 μM MitoSOX Red mitochondrial superoxide indicator (Thermo Fisher Scientific Incorporated) and incubated at 37°C for 10 minutes. After staining, the cells were washed immediately, resuspended in AutoMACS Running Buffer (Miltenyi Biotec), and evaluated using an Attune NxT Flow Cytometer.

### THP-1 Cell Polarization to M1 Macrophages

We harvested the cells 24 hours after LPS administration. The cells were stained with an FITC-anti-human CD80 antibody (BioLegend, San Diego, California, USA) or FITC mouse IgG1, k isotype (Becton, Dickinson and Company, Franklin Lakes, New Jersey, USA) for 30 minutes at 4°C after blocking the nonspecific Fc receptor using FC blocking reagent human (Miltenyi Biotec) for 10 minutes. After staining, the cells were washed immediately and resuspended in AutoMACS Runnig Buffer. Fluorescence data were collected using an Attune NxT Flow Cytometer, and the FITC-positive cell population was evaluated using an excitation wavelength of 488 nm. The flow cytometry files were analyzed using FlowJo software (Becton, Dickinson and Company).

### U937 Cell Polarization to M2 Macrophages

U937 cells were cultured in RPMI 1640 medium supplemented with 10% FBS and 1% penicillin/streptomycin and incubated at 37°C in a humidified 5% CO_2_ incubator. We polarized cells into M2 macrophages by seeding the cells in 6-well cell culture plates (Corning Incorporated) at a density of 1 × 10^6^ cells per well in growth medium containing 100 nM PMA at -48 hours. After 48 hours (at 0 hours), the medium was carefully removed to avoid detaching the cells attached to the bottom of the plate and replaced with new medium containing the appropriate concentration of EEQ-A or DMSO (0.1%) as a negative control and IL-4 (50 ng/ml) as a positive control. After 72 hours (at 72 hours), the cells were harvested. Cells were stained with Alexa Fluor 647-conjugated anti-human CD209 antibody (BioLegend) or Alexa Fluor 647-conjugated mouse IgG2a, k isotype (BioLegend) for 30 minutes at 4°C and blocked with nonspecific Fc receptor using FC Blocking Reagent Human for 10 minutes. After staining, the cells were washed immediately and resuspended in AutoMACS Running Buffer. Fluorescence data were collected using MA900 (SONY, Tokyo, Japan). The Alexa Fluor 647-positive cell population was evaluated using an excitation wavelength of 638 nm. The flow cytometry files were analyzed using FlowJo software.

### Measurements of Respiratory Function

An XFe96 extracellular flux analyzer (Agilent Technologies, Santa Clara, USA) was used to measure cellular respiratory function. The cells were suspended in Seahorse XF RPMI medium (Agilent Technologies) containing 10 mM glucose, 1 mM pyruvate, and 2 mM L-glutamine and seeded on XFe96 well microplates (Agilent Technologies) coated with Cell-Tak (Corning Incorporated) at a density of 1 × 10^5^ cells per well. After seeding, the cells were equilibrated in a non-CO_2_ incubator for 20 minutes and used in the assay. After baseline measurements, oligomycin (2 μM), carbonyl cyanide p-trifluoro methoxyphenyl hydrazone (FCCP, 2 μM) and rotenone/antimycin A (0.5 μM), which were adjusted using the reagents in the Seahorse XF cell Mito Stress Test kit (Agilent Technologies), were sequentially added to each well. The data are presented as the oxygen consumption rate (OCR; pmol/minute). Basal respiration, ATP production, maximal respiration, proton leakage, spare respiratory capacity, non-mitochondrial oxygen (non-MTC) and coupling efficiency were calculated.

### Measurements of Glycolysis

An XFe96 extracellular flux analyzer was used to measure glycolysis. Cells were suspended in Seahorse XF RPMI medium containing 2 mM L-glutamine and seeded on XFe96 well microplates coated with Cell-Tak at a density of 1 × 10^5^ cells per well. After seeding, the cells were equilibrated in a non-CO_2_ incubator for 20 minutes and used in the assay. After baseline measurements, glucose (10 mM), oligomycin (1 μM) and 2-deoxy-D-glucose (2-DG, 50 mM), which were adjusted using the reagents in the Seahorse XF cell glycolysis stress test kit (Agilent Technologies), were sequentially added to each well. The data are presented as the extracellular acidification rate (ECAR; mpH/minute). Glycolysis, glycolytic capacity and glycolytic reserve were calculated.

### Phagocytosis by Macrophages

Phagocytosis was assessed using a Phagocytosis Assay Kit IgG-FITC (Cayman Chemical, Ann Arbor, USA). THP-1 cells suspended at a concentration of 1 × 10^6^ in 1 ml of culture medium were stained with the Latex Beads-rabbit IgG-FITC Complex from the kit for 20 minutes at 37°C. After staining, cells were centrifuged at 400 × G for 5 minutes and resuspended in 200 μl of autoMACS Running Buffer. Phagocytosis was assessed by measuring the MFI of FITC based on cells treated without either EEQ-A or LPS or the FITC positivity rate of unstained cells.

### Lysosomal pH of Macrophages

Lysosomal pH was assessed using pHrodo Green dextran (Cayman Chemical). THP-1 cells suspended at a concentration of 1 × 10^6^ in 1 ml of culture medium were stained with 20 μg/ml pHrodo Green dextran from the kit for 20 minutes at 37°C. After staining, cells were centrifuged at 400 ×g for 5 minutes and resuspended in 200 μl of autoMACS Running Buffer. Using flow cytometry (FCM), lysosomal pH was assessed by determining the MFI of FITC based on cells treated without either EEQ-A or LPS or the FITC positivity rate of unstained cells.

### Statistical Analysis

The results are presented as the means ± standard deviation. The statistical significance of differences among groups was evaluated using parametric unpaired t-tests for bar graphs, one-way ANOVA analysis for the comparison between more than two groups and using two-way ANOVA analysis for time course study. Mantel-Cox tests was used for statistical analysis of data sets of Kaplan-Meier survival curves (Prism 7 software, GraphPad Prism Software Inc., San Diego, CA, USA). P < 0.05 was considered to indicate significance.

## Results

### The Effect of EEQ-A on Endotoxemia Model Mice

**Figure 1:**
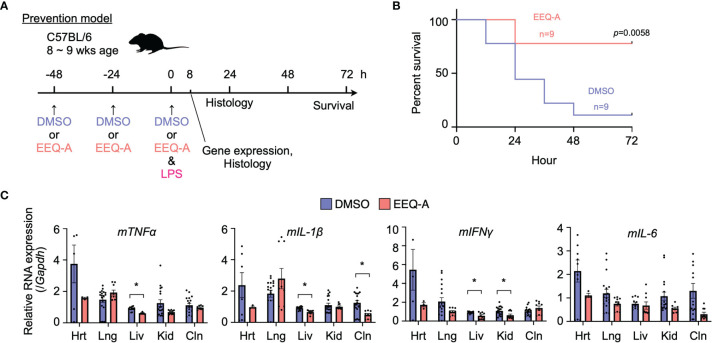
**(A)** Prevention protocol. **(B)** Kaplan–Meier survival curve of mice subjected to the prevention protocol. **(C)** The mRNA expression of proinflammatory cytokines in mice subjected to the prevention protocol at 8 hours after LPS administration. Hrt, Heart; Lng, Lung; Liv, Liver; Kid, Kidney; Cln, Colon. The p-value at 72 hours is shown. *0.0001 to 0.05.

We examined the effect of EEQ-A on an endotoxemia model established by intraperitoneally injecting LPS. Intraperitoneal injection of either EEQ-A or vehicle into a control group every 24 hours for 72 hours beginning at -48 hours was defined as the prevention protocol, and at 0 hours, LPS was injected following the 3rd administration of EEQ-A or vehicle (**Figure 1A**). In the control group, about half of the mice died already by Day 1, and only one of 9 mice survived by 72 hours (**Figure 1B**, **Supplementary Figure 1A**). In contrast, the EEQ-A group lost 2 mice at 24 hours, and the remaining 7 mice survived over the whole observation period, while looking healthy. The Kaplan–Meier plot showed that the survival rate of the EEQ-A group was significantly different from that of the control group at 72 hours (**Figure 1B**). We created another treatment protocol, where LPS was intraperitoneally injected first, and then either EEQ-A or vehicle was administered every 24 hours for 72 hours beginning on the day of LPS injection (**Supplementary Figure 1B**). Mice began to die in both groups after 24 hours, but no significant difference was detected. A large difference in death between the two groups was observed after 36 hours, and their survival was detected as a significant difference after 48 hours. At 72 hours, which was defined as the end of the test, there was a large difference between the two groups, with 10 animals surviving in the experimental group and 5 animals in the control group (**Supplementary Figures 1C, D**). However, the p-value was greater than the 5% level of significance. The experiments were conducted in 3 sessions, all of which showed a similar trend, with the p-value at 72 hours approaching the significance level with the number of the sessions. The reason for the inability to reject the null hypothesis could be a beta error, where the small number means that there is a difference but it cannot be detected. This protocol of intraperitoneal administration without direct clinical relevance is not positioned as a preclinical study. From the viewpoint of animal welfare, we refrained from increasing the number of animals to confirm significant differences.

### EEQ-A Suppressed Proinflammatory Cytokine Production *In Vivo*

In the prevention model, major organs, namely, the heart, lung, liver, kidney, and colon, were harvested 8 hours after the intraperitoneal injection of LPS to examine the mRNA of proinflammatory cytokines, including *TNFα*, *IL-1β*, *IL-6*, and *IFNγ* (**Figure 1C**). At this time point, EEQ-A reduced the transcript levels of the proinflammatory cytokines in the liver, although there were some exceptions. Their mRNA expression levels in the EEQ-A group were lower than those in the control group except for the lung, whereas no significant differences except for *IFNγ* were observed in the right kidney. In particular, the heart exhibited a large difference in the average values for all cytokines examined between groups. The difference among organs with respect to responsiveness to EEQ-A might indicate that micro-environment consisting of various immune cells, including tissue-resident macrophages, were differentially regulated following the LPS insult (**Figure 1C**).

### EEQ-A Prevented Hyperactivated Immune Responses Induced by LPS

**Figure 2:**
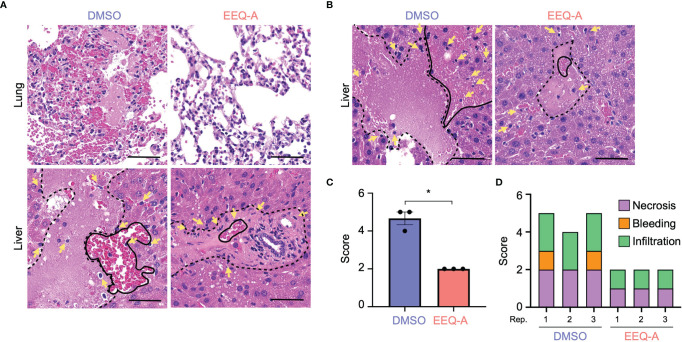
**(A)** Lung and liver of mice subjected to the prevention protocol at 8 hours after LPS administration. Yellow arrows show infiltrating cells. The area surrounded by the dotted line is necrosis. The area surrounded by the solid line is bleeding. **(B)** The liver of mice subjected to the prevention protocol at 24 hours after LPS administration. Yellow arrows show infiltrating cells. The area surrounded by the dotted line is necrosis. The area surrounded by the solid line is bleeding. **(C)** The total inflammatory score of the liver in mice subjected to the prevention protocol at 24 hours after LPS administration (n = 3). **(D)** The categories of the liver inflammatory score (n = 3). *0.0001 to 0.05. One image was taken per organ of each animal. All bars on the images are 50 μm.

Major organs, namely, the heart, lung, liver, kidney, and colon, were harvested 8 and 24 hours after LPS injection in the prevention model to investigate pathological changes. In the early phase at 8 hours after injection, microvascular bleeding and infiltration were recognized in all organs, the extent of which was worst in the lungs of the control group, in contrast to the EEQ-A group, which showed moderate bleeding, destruction of alveolar structure and protein leakage (**Figure 2A**). The hearts, kidneys and colons displayed little bleeding and infiltration in both the EEQ-A and control groups, without any differences (**Supplementary Figure 2A**). Liver damage was scored and did not show a significant difference at 8 hours following LPS infusion, although infiltrations initiated without bleeding and necrosis were observed in both groups (**Figure 2A**, **Supplementary Figure 2B**). Twenty-four hours following the LPS injection, the livers of the control group showed substantial necrosis with bleeding and infiltrations, scoring >4 on average. On the other hand, the EEQ-A group did not exhibit necrosis but showed mild bleeding and infiltration, scoring 2 on average (**Figures 2B, C**). Damage, such as infiltration, bleeding, and necrosis, was quantified and integrated into a score (**Figure 2D**). These histopathological findings revealed the efficacy of EEQ-A at preventing hyperactivated immune responses induced by LPS in various organs, rather than restricted organs or tissues. Based on the animal experiments, we decided to investigate the mode of action of EEQ-A *in vitro* through LPS stimulation following EEQ-A exposure.

### EEQ-A Suppresses the Transcript Level of pro-Inflammatory Cytokines and Increases the Transcript Level of Anti-Inflammatory Cytokines

**Figure 3:**
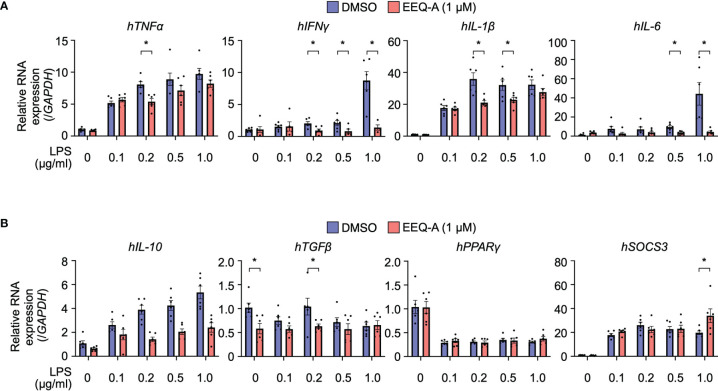
RNA expression of proinflammatory **(A)** and anti-inflammatory **(B)** cytokines in THP-1 cells, the inflammation model. The cells were harvested 3 hours after LPS administration. P-values *0.0001 to 0.05. All experiments were triplicated.

We elucidated the mode of action by which EEQ-A ameliorates LPS-induced hyperactivation of innate immunity by examining cytokine transcript level profiles using THP-1 cells, which are human monocytic cells derived from acute monocytic leukemia and are widely used for macrophage differentiation models (32). Based on previous reports (33), we developed a protocol in which THP-1 cells were preconditioned to express CD14 by administering 10 nM PMA. Proinflammatory and anti-inflammatory cytokine transcript levels were estimated in a protocol simulating the *in vivo* prevention protocol. Additionally, the dose dependency of LPS was examined with concentrations ranging from 0.1 to 1.0 μg/ml. At 3 hours after LPS administration, the transcript levels of all pro-inflammatory cytokines, especially *TNFα* and *IL-1b*, increased by LPS administration (**Figure 3A**). The transcript levels of *TNFα* in cells with 0.2 μg/ml LPS, *INFγ* in cells with 0.2, 0.5, and 1.0 μg/ml, *IL-1β* in cells with 0.2 and 0.5 μg/ml LPS and *IL-6* in cells with 0.5 and 1.0 μg/ml LPS was significantly decreased by EEQ-A administration. The transcript levels of these pro-inflammatory cytokines showed a decreasing trend with longer LPS administration time (6, 12, 24 hours), and was considered to reach its peak transcript level in a relatively short time (**Supplementary Figure 3**). Even at 24 hours after LPS administration, the tendency of suppression of the transcript levels of pro-inflammatory cytokines by EEQ-A was observed. The transcript levels of anti-inflammatory cytokines increase only in *IL-10* and *SOCS3* in 3 hours after LPS administration (**Figure 3B**). Only transcript level of *SOCS3* with LPS 1.0 μg/ml significantly increased by EEQ-A administration. The transcript levels of *IL-10* and *TGFβ* trended to decrease with EEQ-A administration, however, no significant difference was observed. The transcript level of *IL-10* peaked at 6 hours after LPS administration and then decreased and there was a trend toward decreased transcript level by EEQ-A at 24 hours (**Supplementary Figure 4**). The transcript level of *SOCS3* peaked at 3 hours after LPS administration and decreased over time. There was no marked increase or suppression by EEQ-A during the time course of the study. The transcript level of *TGFβ* and *PPARγ* was not obviously increased during the 24 hours after LPS administration.

### EEQ-A Inhibits Macrophage Differentiation Into the M1 Phenotype and Promotes Macrophage Differentiation Into the M2 Phenotype

**Figure 4:**
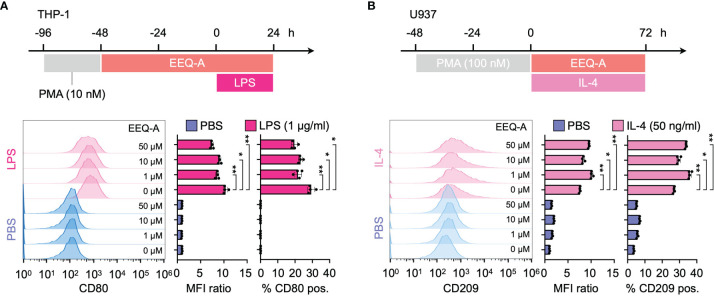
**(A)** Polarization protocol to M1 macrophages by THP-1 cells. The histogram shows the MFI ratio of FITC for anti-human CD80 antibodies and the percentage of M1 macrophages. **(B)** The polarization protocol to M2 macrophages by U937 cells. The histogram shows the MFI ratio of Alexa Fluor 647 for anti-human CD209 antibodies and the percentage of M2 macrophages. **: < 0.0001, *: 0.0001 to 0.05. All experiments were triplicated.

Using a previous protocol designed to differentiate THP-1 or U937 cells into either the M1 or M2 phenotype of macrophages, we evaluated the effect of EEQ-A on those differentiation processes by measuring CD80 or CD209 expression, which are biological markers for the M1 or M2 phenotype, respectively. LPS stimulation substantially increased the expression of CD80, a marker of the M1 phenotype, and exposure to EEQ-A significantly suppressed CD80 expression (**Figure 4A** and **Supplementary Figure 5A**). U937 cells, a myeloid cell line used as an M2 polarization model, showed significantly increased expression of CD209, a marker of the M2 phenotype of macrophages, following IL-4 administration and this effect was further enhanced by EEQ-A (**Figure 4B** and **Supplementary Figure 5B**). Taken together, these data in human macrophage differentiation models indicate that EEQ-A is able to repress M1 polarization and to promote M2 polarization, in line with its anti-inflammatory properties.

Phagocytic capabilities and lysosomal degradation are essential functions of macrophages and influence outcomes upon infection. Regarding macrophage characteristics, either phagocytosis or lysosomal pH on THP-1 cells was evaluated after treatment with increasing concentrations of LPS ranging from 0.01 to 1 μg/ml to examine whether EEQ- A modulates this process (**Supplementary Figure 6**). Phagocytosis was not affected by any concentration of LPS in cells treated with EEQ- A. Also, lysosomal pH was not changed by EEQ- A, regardless of the LPS concentration applied, except for the mean fluorescence intensity (MFI) and lysosomal positive rate observed at 1 and 0.01 μg/ml, respectively, suggesting that these two processes are not intertwined with the mechanism of action of EEQ-A in regulating the LPS-induced pathology. We hypothesized that metabolism influences macrophage polarization.

### EEQ-A Modifies Mitochondrial Functions Upon LPS Stimulation

**Figure 5:**
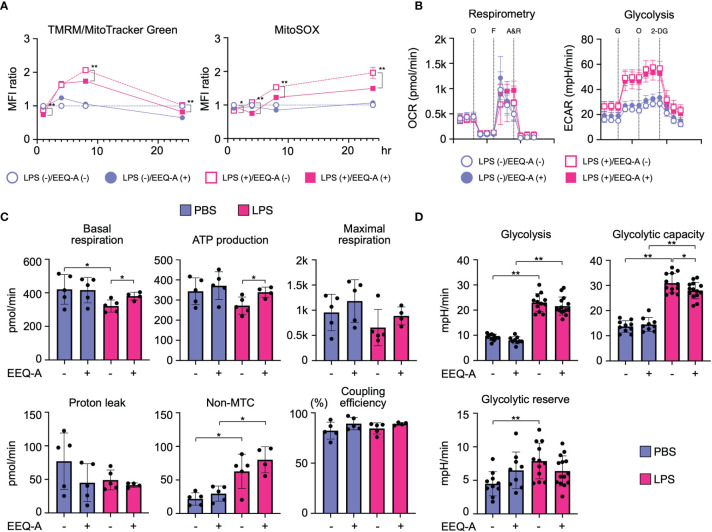
**(A)** MFI of Δφ and mtROS levels in THP-1 cells, the inflammation model, 1, 4, 8, and 24 hours after LPS administration. All data were evaluated using unpaired t test only between closed and open circles or squares. **(B)** Changes in the OCR and ECAR of inflammation model THP-1 cells 24 hours after LPS administration, as measured using flux analysis. O: oligomycin, F: FCCP, A&R: antimycin and rotenone, G: glucose, 2-DG: 2-deoxy-D-glucose. **(C)** Each index of mitochondrial respiratory function was calculated from the results of the OCR measurement. **(D)** Each index of glycolysis was calculated from the results of the ECAR measurement. **: < 0.0001, *: 0.0001 to 0.05. All experiments were triplicated.

Because PUFAs and their metabolites have been shown to modify mitochondrial function, we examined whether EEQ-A influences mitochondrial functions under the conditions of LPS-induced M1 polarization. The mitochondrial membrane potential (Δφ) temporally increased following LPS stimulation, whereas EEQ-A suppressed the increase in Δφ (**Figure 5A**, left panel and **Supplementary Figure 7**, left panel). Mitochondrial reactive oxygen species (mtROS) levels were increased upon LPS stimulation and decreased in response to EEQ-A (**Figure 5A**, right panel and **Supplementary Figure 7**, right panel). Furthermore, we analyzed metabolic changes induced by EEQ-A by measuring oxygen consumption rates (OCRs) and extracellular acidification rates (ECARs) using a flux analyzer. ATP generation and basal respiration were significantly increased upon EEQ-A administration following LPS stimulation (**Figures 5B**, left panel and **5C**). Although no significant differences in proton leakage, maximum respiration, and coupling efficiency were observed between the EEQ-A and control groups, all parameters exhibited increasing trends. Collectively, these EEQ-A-induced alterations in mitochondrial function suggested the compaction of the respiratory supercomplex, despite the lack of direct structural evidence available in this study. In addition, we assessed glycolysis by measuring ECARs (**Figures 5B** right and **5D**). Glycolysis and the glycolytic capacity were significantly increased upon LPS stimulation, and EEQ-A administration significantly decreased the LPS-induced increase in the glycolytic capacity.

## Discussion

Preventive administration of the synthetic Omega-3 epoxyeicosanoid analog EEQ-A significantly improved the survival rate, reduced organ damage, and suppressed pro-inflammatory cytokine expression in our mouse model of LPS-induced endotoxemia. As further analyzed *in vitro*, the potential mode of action of EEQ-A includes immunomodulatory effects on macrophage function comprising repression of LPS-induced mitochondrial dysfunction and M1 polarization as well as stimulation of M2 polarization. These results indicate the ability of EEQ-A to target important mechanisms leading to the endotoxemia and suggest that Omega-3 epoxyeicosanoid analogs may provide novel therapeutic options for the treatment of acute inflammatory disorders.

**Figure 6:**
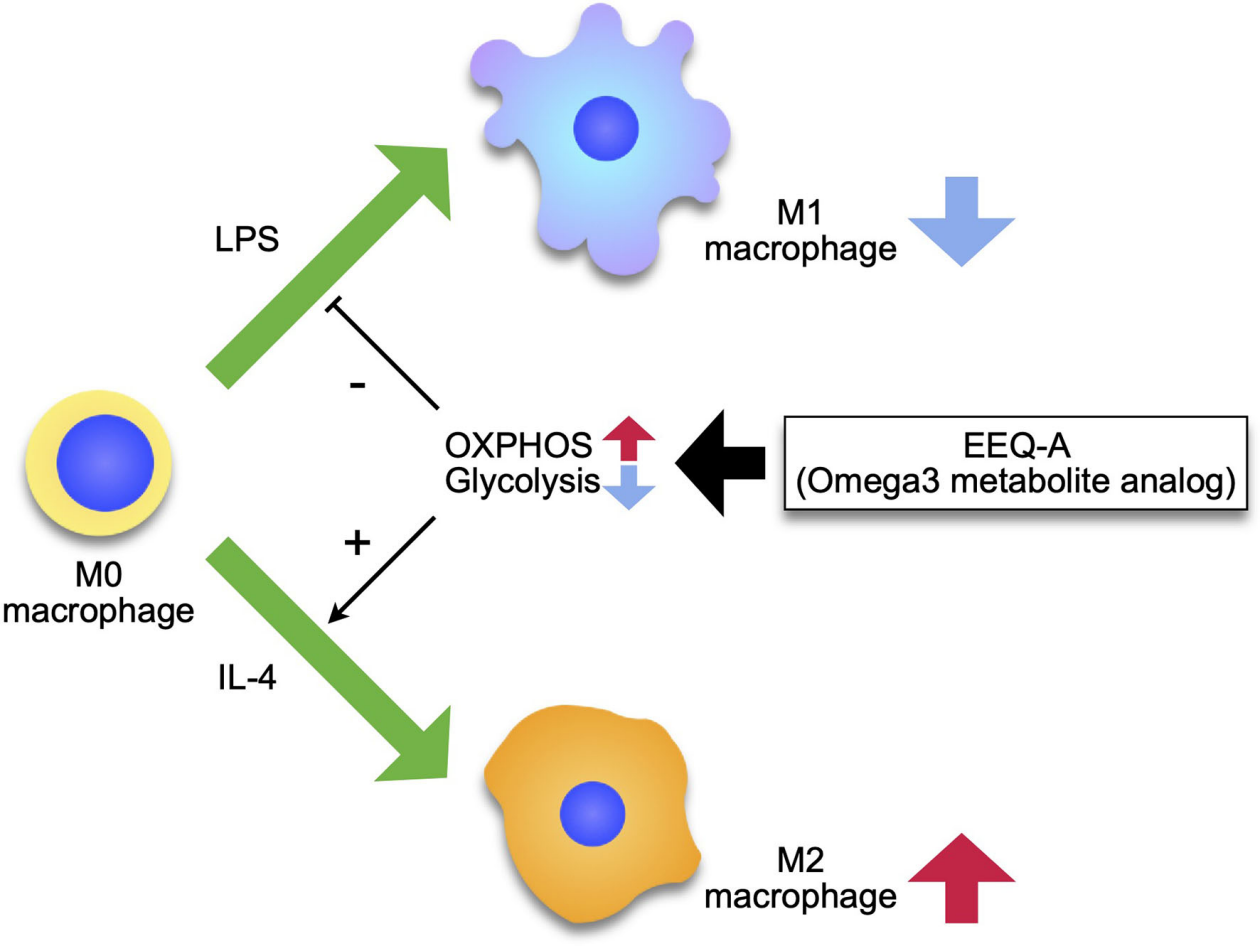
Model of the effect of EEQ-A on macrophages. Macrophages differentiate into M1 or M2 by external stimuli, and this process is regulated by metabolism, with M1 depending on the glycolysis for energy and M2 being maintained by the mitochondrial respiratory chain activity. EEQ-A has a metabolism-targeted mode of action, suppressing the glycolysis and enhancing OXPHOS, thereby inhibiting differentiation to M1 and promoting differentiation to M2.

The signal transduced by the engagement of LPS to TLR4 is mediated to Akt *via* phosphatidyl inositol 3 kinase (PI3K), leading to increased expression of GLUT1 to ensure an adequate glucose supply (34). Naïve macrophages physiologically utilize glucose as a substrate for oxidative phosphorylation (OXPHOS) to acquire energy rather than glycolysis (35), whereas M1 macrophages shift the metabolic pathway from OXPHOS to glycolysis by increasing fatty acid synthesis (FAS) (36). FAS supplies the compartment of the ER and Golgi apparatus to generate proinflammatory cytokines and chemokines, and augments phagocytosis by supplying membrane components (37). LPS disrupts the Krebs cycle by inhibiting icocitrate dehydrogenase and succinate dehydrogenase. In individuals with sepsis, levels of two intermediates of the TCA cycle in mitochondria, citrate and succinate, are significantly increased (38). The accumulation of citrates in the cytosol is induced through the expression of mitochondrial citrate carrier (solute carrier family 25 member 1: Slc215a1) by NF-κB at the earlier phase before the surge of nitric oxide (NO), which is generated in the early phase of LPS administration (39), and then strengthened by the inhibition of aconitase by NO (40). Citrates to be imported to the cytosol from mitochondria are used as donors of NO and ROS through NADPH and FAS. NO contributes to the inhibition of OXPHOS by nitrosylating iron-sulfur proteins, which are contained in respiratory chain complex I and cytochrome c oxidase (41). Another intermediate, succinates, is exported out of mitochondria through glutaminolysis through a mechanism called anaplerosis (42), and inhibits prolyl hydroxylase, which degrades HIF-1α, driving inflammation (43). M2 macrophages mainly use fatty acids as substrates for mitochondrial OXPHOS (44). The plasticity of macrophages is enhanced in the M2 type, which are repolarized to M1, while M1 macrophages are prevented from repolarizing to the M2 type following mitochondrial OXPHOS dysfunction induced by nitric oxide, which is promptly induced in sepsis (45). Researchers proposed that pharmacological interventions targeting macrophage metabolism overcome the hurdle of the class switch from M1- to M2-type macrophages using AMPK activators (46). In the early phase of sepsis, attenuation of M1 and/or enhancement of M2 macrophages shows therapeutic potential to prevent excessive inflammation. EEQ-A decreased the differentiation to the M1 phenotype and increased the polarization to M2 phenotype, therefore, if translatable to the *in vivo* situation, the ratio of M2/M1 should be considerably enhanced by EEQ-A. In T cell fate analyses based on metabolism, OXPHOS supports the differentiation to memory type, whereas glycolysis deviated T cells to effector phenotype (47). In macrophages, there are some reports indicating similar metabolic regulation (48). EEQ-A promoted mitochondrial respiration and reduced glycolysis capability in macrophages. These characteristics of EEQ-A might have contributed to its pro-survival effect in the LPS models, since macrophages are essential to the pathology in this LPS-induced endotoxemia model (49). We propose the mode of action of EEQ-A as **Figure 6** depicts.

For a long time, there was no clear concept of the mechanism that resolves the inflammatory response triggered by infection. Recently, however, it has become recognized that there are mediators, called specialized pro-resolving mediators (SPMs), that convert inflammation into resolution. Resolvin, protectin, and maresin are known as SPMs, all of which are derived from Omega-3 fatty acids (50). SPMs affect the intensity and duration of inflammation and survival in animal models (51, 52) and could be useful in predicting clinical outcomes (53). While the treatment of inflammatory diseases has mainly been to suppress the activation of pro-inflammatory responses, targeting SPMs to promote the resolution of inflammation could be an alternative therapeutic strategy. Although EEQ-A isn’t a direct analog of SPMs, the mode of action is in line with SPMs, which calm down the inflame of the activated immunity. This study suggested that lipid mediators play an essential role in not only chronic cardiovascular disorders (6), but also acute inflammatory diseases.

The results of the present study are in line with an important role of the CYP epoxygenase pathway of eicosanoid formation in regulating inflammatory responses. The CYP-derived epoxyeicosanoids and, in particular those generated from n-3 PUFAs, have been identified as anti-inflammatory and cytoprotective metabolites (6, 10, 12). Their endogenous levels largely depend on the bioavailability of the required PUFA precursors, the expression and metabolic activity of CYP epoxygenases, and the rate of sEH-mediated degradation (6). Furthermore, inflammation might specifically limit the formation and protective action of epoxyeicosanoids by down-regulating CYP and up-regulating sEH expression (54–59). In line with this notion, measures to increase endogenous epoxyeicosanoid levels, such as overexpression of CYP epoxygenases and sEH-inhibition, exerted protective effects in mouse models of LPS-induced septic shock (13). In clinical situations, increased generation of Omega-3 epoxyeicosanoids can be achieved through EPA/DHA-supplementation (19, 60);however, the individual responses may be affected by background nutrition containing competing n-6 PUFAs, disease-related changes in relative CYP/sEH expression (6), and functional polymorphisms in the CYP and sEH genes (61, 62). Principal approaches to overcome these uncertainties and potential limitations are under development and include (i) a combination of n-3 PUFA supplementation with the administration of sEH inhibitors and (ii) the use of metabolically robust Omega-3 epoxyeicosanoids (6). The first approach prevents sEH-mediated hydrolysis of virtually all epoxyeicosanoids as endogenously produced from various PUFAs, but targets neither their CYP epoxygenase-dependent generation nor sEH-independent pathways of their further metabolism, such as oxygenation by COX and LOX enzymes or hydrolysis by microsomal epoxide hydrolases (6, 63). Furthermore, reduction of potentially detrimental vicinal diol production may contribute to the observed beneficial effects of sEH –inhibitors (15, 64). The second approach is aimed at specifically compensating deficiencies in endogenous Omega-3 epoxyeicosanoid levels by supplementing a metabolically robust synthetic analog that mimics their biological activities. This approach is likely much less dependent on the host environment compared to n-3 PUFA-supplementation and sEH-inhibition.

With the goal of developing an effective small molecule to ameliorate the pathophysiology of LPS-induced endotoxemia, this experiment has been designed to examine whether Omega-3 analog can intervene in inflammatory responses, and what the mode of action is. We found that Omega-3 analog successfully improved the survival of mice suffered from endotoxemia, and the modification of mitochondrial function could be a mode of action. This study provided the foundation for the next stage of drug discovery. The most fundamental limitation of this study is the validity of LPS-injected mice as a clinical model. CLP is the most widely used model of sepsis in recent years and was reported to have a more realistic phenotype than the LPS-based model (65, 66). CLP requires laparotomy, ligating a portion of the cecum, and piercing the cecum with a needle (67), which may result in different outcome depending on the operator. Although inappropriate for preclinical studies in drug discovery for sepsis, the LPS model is still important for analyzing the mechanism of inflammation and investigating the mode of action of compounds for early phase of drug discovery for inflammatory disorders (68).

In this study, we showed that EEQ-A is effective for early inflammatory reactions, which could be executed through metabolic intervention to mitochondria in macrophages.

## Data Availability Statement

The original contributions presented in the study are included in the article/**Supplementary Material**. Further inquiries can be directed to the corresponding author.

## Ethics Statement

The animal study was reviewed and approved by The Animal Experiment Ethics Committee of the Kyoto Prefectural University of Medicine (approval number M2021-547).

## Author Contributions

SG designed the experiments, analyzed the data, and wrote the manuscript. ASh performed the experiments and analyzed the data with DK. RM, YS, ASa, TT, and TO analyzed and discussed the data. SM discussed the clinical relevance. W-HS and AK supervised the use of EEQ-A and contributed to the preparation of the manuscript. All authors contributed to the article and approved the submitted version.

## Funding

The authors declare that this study was supported by funding from MEDINET Co., Ltd. (Tokyo, Japan). The funder was not involved in the study design, collection, analysis, interpretation of data, the writing of this article or the decision to submit it for publication.

## Conflict of Interest

SG and DK received a collaborative research grant from OMEICOS Therapeutics GmbH for another research theme apart from this study. W-HS and AK are a founder and an employee of OMEICOS Therapeutics GmbH, respectively, and are not involved in the design, analysis, or interpretation of this study.

The remaining authors declare that the research was conducted in the absence of any commercial or financial relationships that could be construed as a potential conflict of interest.

## Publisher’s Note

All claims expressed in this article are solely those of the authors and do not necessarily represent those of their affiliated organizations, or those of the publisher, the editors and the reviewers. Any product that may be evaluated in this article, or claim that may be made by its manufacturer, is not guaranteed or endorsed by the publisher.

## Supplementary Material

Supplementary Figure 1
**(A)** Kaplan-Meier survival curve by gender in the prevention model. The p-value at 72 hours is shown. **(B)** Treatment protocol **(C)** Kaplan–Meier survival curve in the treatment model. **(D)** Kaplan-Meier survival curve by gender in the treatment model. The p-value at 48 and 72 hours is shown.

Supplementary Figure 2
**(A)** The heart, right kidney and colon tissue from mice subjected to the prevention protocol at 8 hours after LPS administration. **(B)** The total inflammatory score and categorization of the score of the livers in mice subjected to the prevention protocol 8 hours after LPS administration. All bars on the images are 50 μm.

Supplementary Figure 3
**|** RNA expression of proinflammatory cytokines in THP-1 cells in inflammation model. The cells were harvested 6, 12, 24 hours after LPS administration. P-values **: < 0.0001, *: 0.0001 to 0.05. All experiments were triplicated.

Supplementary Figure 4 RNA expression of anti-inflammatory cytokines in THP-1 cells in inflammation model. The cells were harvested 6, 12, 24 hours after LPS administration. P-values **: < 0.0001, *: 0.0001 to 0.05. All experiments were triplicated.

Supplementary Figure 5
**(A)** The histogram of inflammation model THP-1 cells polarized to M1 macrophages. **(B)** The histogram of U937 cells polarized to M2 macrophages.

Supplementary Figure 6
**(A, B)** The assessment of phagocytosis (A) and lysosomal pH **(B)** of THP-1 cells, the inflammation model. The MFI ratio of FITC to a Phagocytosis Assay Kit IgG-FITC (A, left panel) and the positive percentage of phagocytosis (A, right panel). The MFI ratio of FITC to pHrodo Green dextran (B, left panel) and the positive percentage of lysosomal pH (**B**, right panel). **(C)** The histogram of phagocytosis and lysosomal pH in the inflammation model THP-1 cells. **: < 0.0001, *: 0.0001 to 0.05.

Supplementary Figure 7 The histogram of Δφ and mtROS levels in the inflammation model THP-1 cells.
