# Supplementary figures and images for: Amelioration of Endotoxemia by a Synthetic Analog of Omega-3 Epoxyeicosanoids
Source: Front Immunol. 2022 Feb 24;13:825171. doi: 10.3389/fimmu.2022.825171 (PMC8908263; doi:10.3389/fimmu.2022.825171)

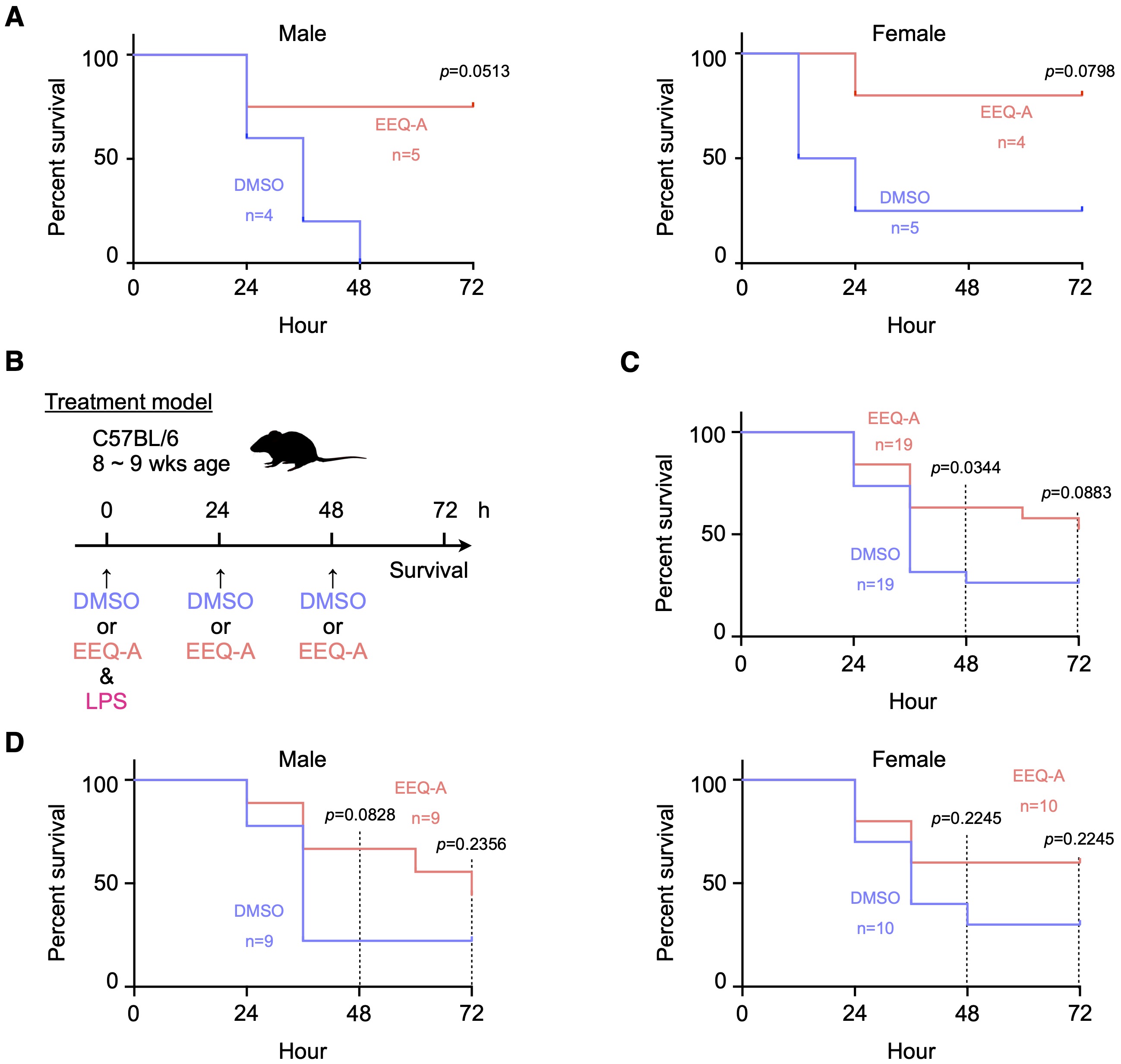

Supplement: Supplementary Figure 1 — (A) Kaplan-Meier survival curve by gender in the prevention model. The p-value at 72 hours is shown. (B) Treatment protocol (C) Kaplan–Meier survival curve in the treatment model. (D) Kaplan-Meier survival curve by gender in the treatment model. The p-value at 48 and 72 hours is shown. [file Image_1.jpeg]

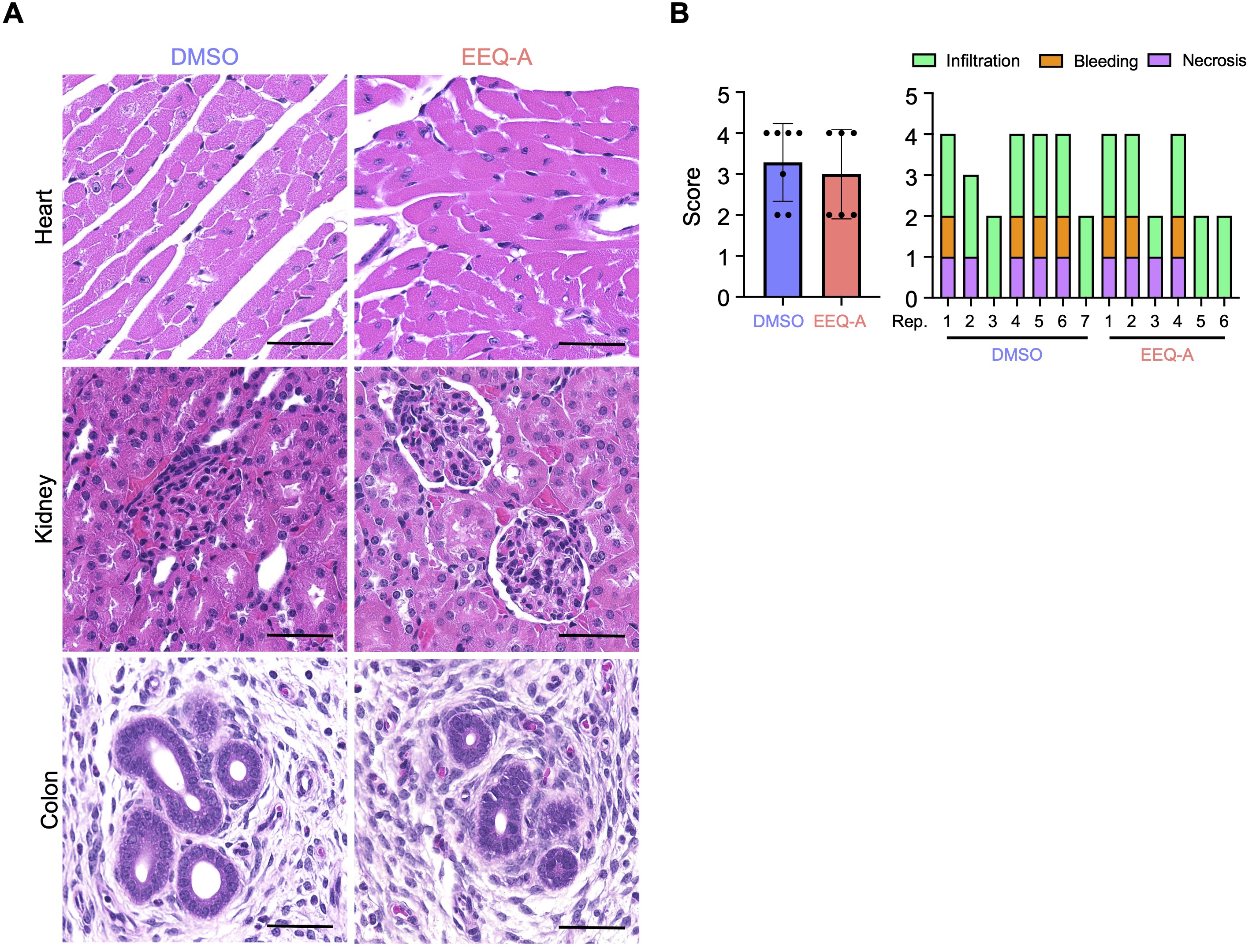

Supplement: Supplementary Figure 2 — (A) The heart, right kidney and colon tissue from mice subjected to the prevention protocol at 8 hours after LPS administration. (B) The total inflammatory score and categorization of the score of the livers in mice subjected to the prevention protocol 8 hours after LPS administration. All bars on the images are 50 μm. [file Image_2.jpeg]

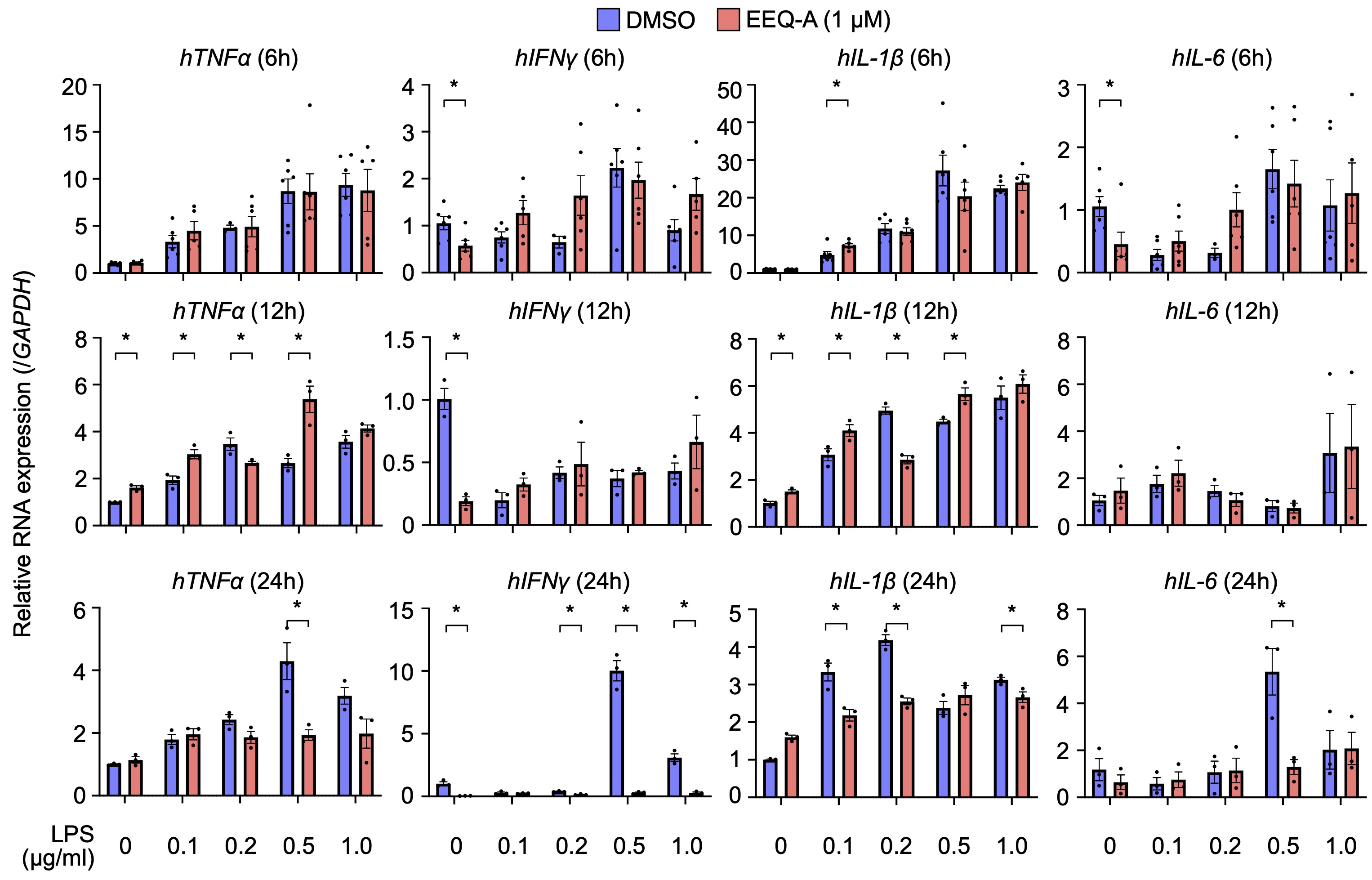

Supplement: Supplementary Figure 3 — | RNA expression of proinflammatory cytokines in THP-1 cells in inflammation model. The cells were harvested 6, 12, 24 hours after LPS administration. P-values **: < 0.0001, *: 0.0001 to 0.05. All experiments were triplicated. [file Image_3.jpeg]

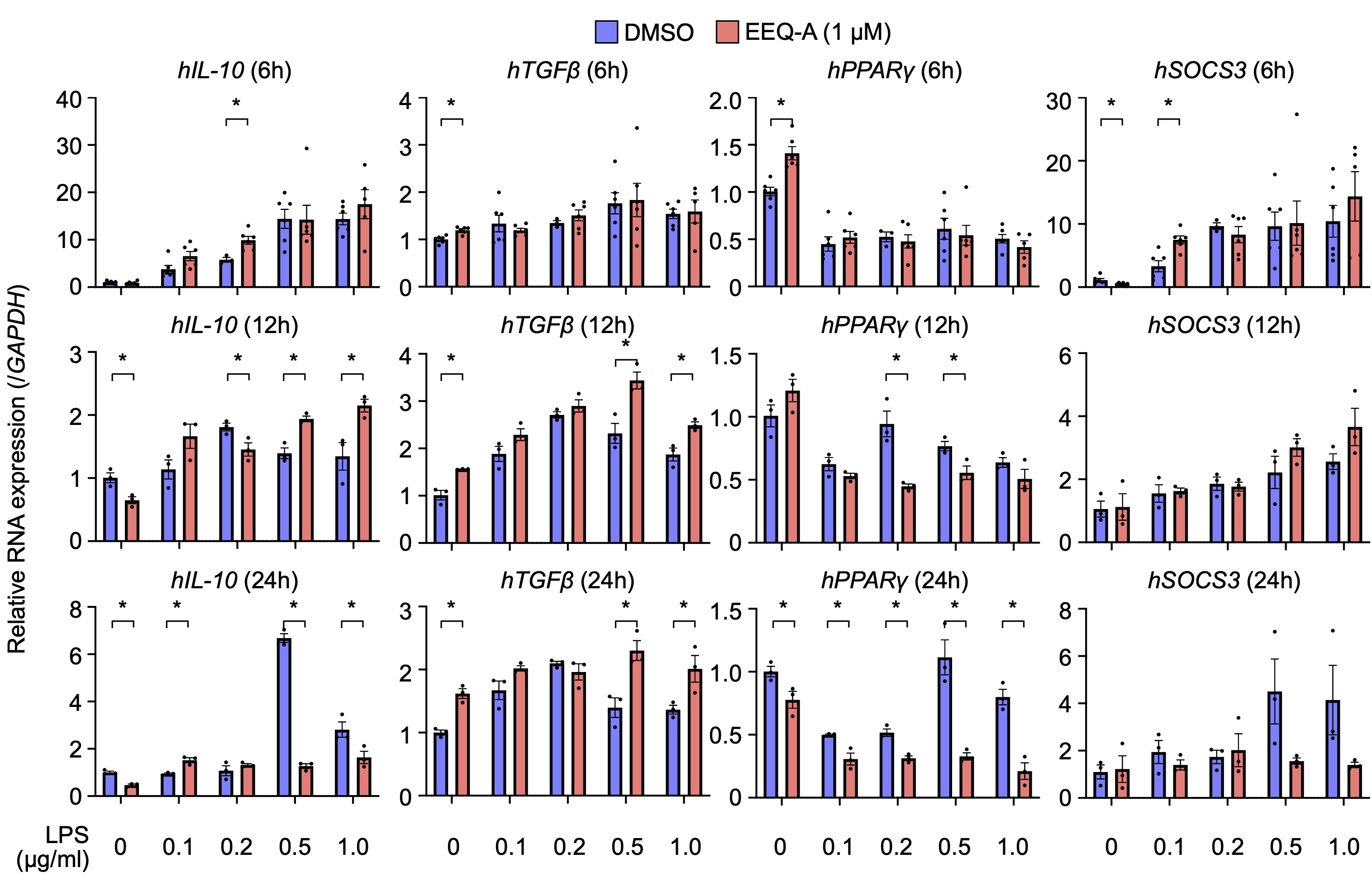

Supplement: Supplementary Figure 4 — RNA expression of anti-inflammatory cytokines in THP-1 cells in inflammation model. The cells were harvested 6, 12, 24 hours after LPS administration. P-values **: < 0.0001, *: 0.0001 to 0.05. All experiments were triplicated. [file Image_4.jpeg]

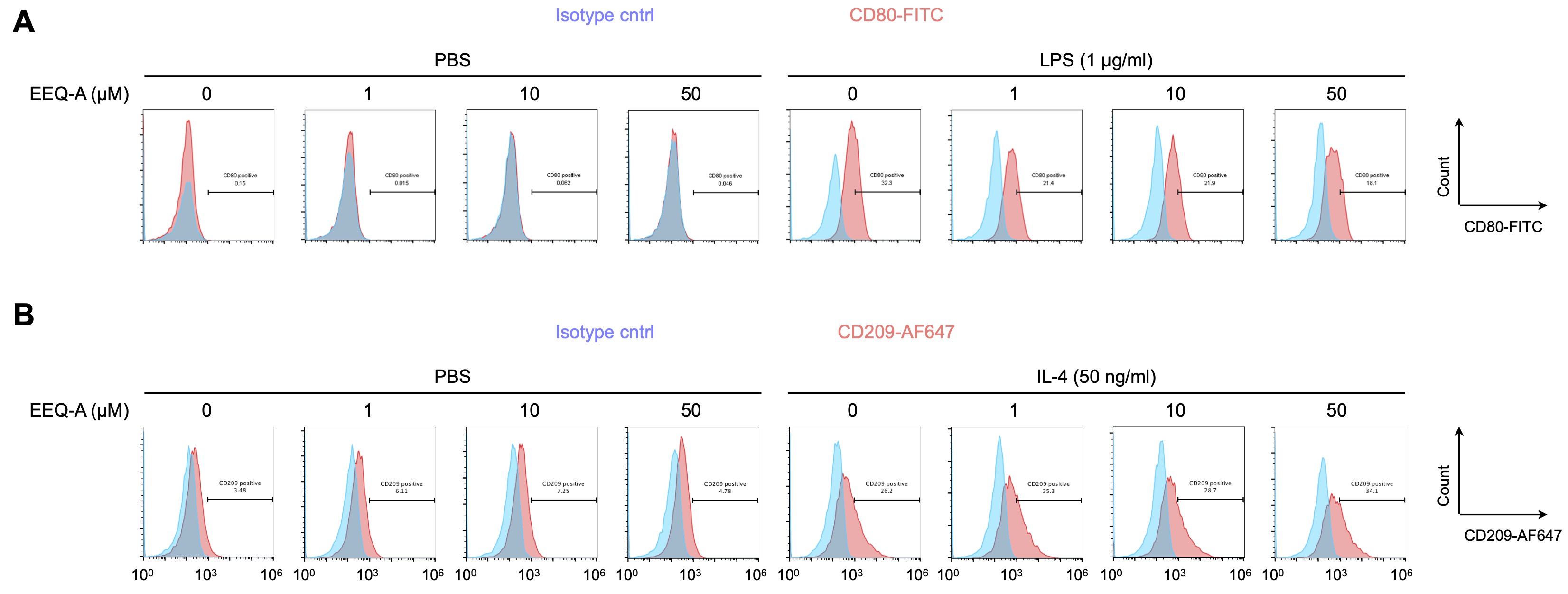

Supplement: Supplementary Figure 5 — (A) The histogram of inflammation model THP-1 cells polarized to M1 macrophages. (B) The histogram of U937 cells polarized to M2 macrophages. [file Image_5.jpeg]

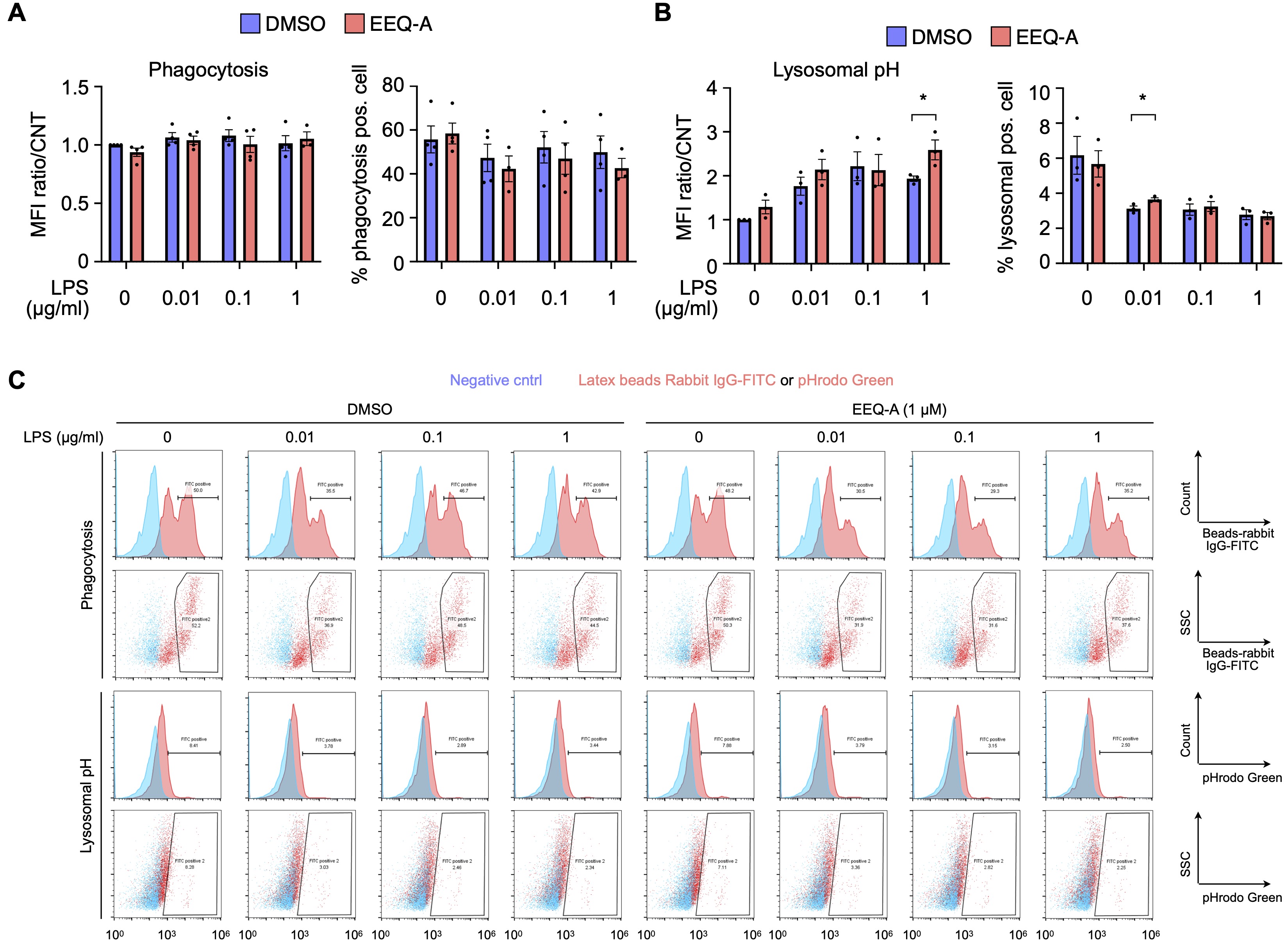

Supplement: Supplementary Figure 6 — (A, B) The assessment of phagocytosis (A) and lysosomal pH (B) of THP-1 cells, the inflammation model. The MFI ratio of FITC to a Phagocytosis Assay Kit IgG-FITC (A, left panel) and the positive percentage of phagocytosis (A, right panel). The MFI ratio of FITC to pHrodo Green dextran (B, left panel) and the positive percentage of lysosomal pH (B, right panel). (C) The histogram of phagocytosis and lysosomal pH in the inflammation model THP-1 cells. **: < 0.0001, *: 0.0001 to 0.05. [file Image_6.jpeg]

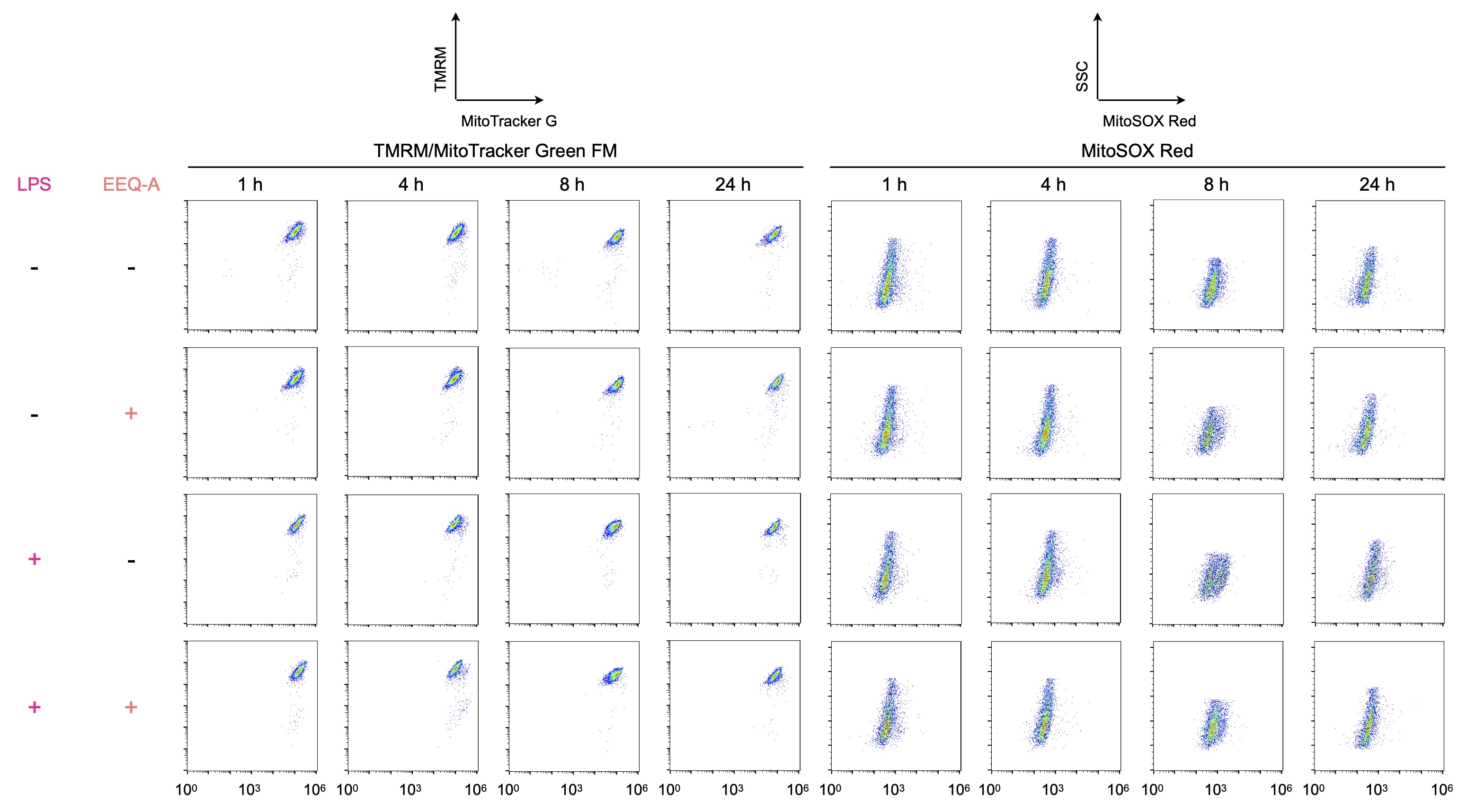

Supplement: Supplementary Figure 7 — The histogram of Δφ and mtROS levels in the inflammation model THP-1 cells. [file Image_7.jpeg]
