# Supplementary material for: Amelioration of Endotoxemia by a Synthetic Analog of Omega-3 Epoxyeicosanoids
Source: Front Immunol. 2022 Feb 24;13:825171. doi: 10.3389/fimmu.2022.825171 (PMC8908263; doi:10.3389/fimmu.2022.825171)
Supplement: Supplementary file 8 [file Table_1.docx]

**Supplement Table 1 |** Primer sequences, the forward and reverse primer sequences were as follows:

| Human | Forward | Reverse |
| --- | --- | --- |
| *TNFα* | 5′‐GAGGCCAAGCCCTGGTATG‐3′ | 5′‐CGGGCCGATTGATCTCAGC‐3′ |
| *IFNγ* | 5′‐TCGGTAACTGACTTGAATGTCCA‐3′ | 5′‐TCGCTTCCCTGTTTTAGCTGC‐3′ |
| *IL-1β* | 5′‐AGCTACGAATCTCCGACCAC‐3′ | 5′‐CGTTATCCCATGTGTCGAAGAA‐3′ |
| *IL-6* | 5′‐ACTCACCTCTTCAGAACGAATTG‐3′ | 5′‐CCATCTTTGGAAGGTTCAGGTTG‐3′ |
| *IL-10* | 5′‐GACTTTAAGGGTTACCTGGGTTG‐3′ | 5′‐TCACATGCGCCTTGATGTCTG‐3′ |
| *TGFβ* | 5′‐CTAATGGTGGAAACCCACAACG‐3′ | 5′‐TATCGCCAGGAATTGTTGCTG‐3′ |
| *PPARγ* | 5′‐GGGATCAGCTCCGTGGATCT‐3′ | 5′‐TGCACTTTGGTACTCTTGAAGTT‐3′ |
| *SOCS3* | 5′‐CCTGCGCCTCAAGACCTTC‐3′ | 5′‐GTCACTGCGCTCCAGTAGAA‐3′ |
| *GAPDH* | 5’-GCTCAGACACCATGGGGAAGGT-3’ | 5’-GTGGTGCAGGAGGCATTGCTGA-3’ |

| Mouse | Forward | Reverse |
| --- | --- | --- |
| *Tnfα* | 5′‐CAGGCGGTGCCTATGTCTC‐3′ | 5′‐CGATCACCCCGAAGTTCAGTAG‐3′ |
| *Ifnγ* | 5′‐GCCACGGCACAGTCATTGA‐3′ | 5′‐TGCTGATGGCCTGATTGTCTT‐3′ |
| *Il-1β* | 5′‐TTCAGGCAGGCAGTATCACTC‐3′ | 5′‐GAAGGTCCACGGGAAAGACAC‐3′ |
| *Il-6* | 5′‐CTGCAAGAGACTTCCATCCAG‐3′ | 5′‐AGTGGTATAGACAGGTCTGTTGG‐3′ |
| *Gapdh* | 5’-TGCGACTTCAACAGCAACTC-3’ | 5’-CTTGCTCAGTGTCCTTGCTG-3’ |
